# Estimation of combined treatment effects by restricted mean survival time

**DOI:** 10.1186/s13063-026-09666-8

**Published:** 2026-04-01

**Authors:** Emily Alger, David S. Robertson, Abigail J. Burdon

**Affiliations:** 1 Clinical Trial and Statistics Unit, Institute of Cancer Research, 15 Cotswold Road, London, SM2 5NG London UK; 2 MRC Biostatistics Unit, University of Cambridge, Forvie Site, Cambridge, CB2 0SR Cambridge UK

**Keywords:** Non-parametric statistics, Proportional hazards, Restricted mean survival time, Survival analysis, Time-to-event

## Abstract

**Background:**

Restricted mean survival time (RMST) endpoints are becoming commonly used as trialists look to analyse time-to-event outcomes without the restrictions of the proportional hazards assumption. An additional benefit of RMST endpoints which has so far remained unexplored is their capability to combine treatment main effects and treatment-by-covariate interaction terms into single one-dimensional estimators under both proportional and non-proportional hazards. By utilising RMST estimators, trialists may assess treatment effects associated with multiple covariates, including interaction terms — an inherent limitation of proportional hazards models when this assumption is violated.

**Methods:**

We present a simulation study using a case study of a randomised controlled trial of Gamma interferon for the treatment of chronic granulomatous disease. We evaluate the power and type I error rate of parametric and non-parametric RMST estimators of combined treatment effects under both proportional and non-proportional hazards. Performance is evaluated when the model or the knot point is specified correctly or misspecified. We also explore the effect of truncation time.

**Results:**

Simulations show that parametric RMST estimators offer greater power when covariate effects and knot-point locations are correctly specified or only mildly misspecified. However, their performance deteriorates as omitted covariate effects increase or knot locations become more misspecified. Under substantial misspecification, the non-parametric estimator is more robust, maintaining stable type I error rates and improved power. For the non-parametric approach, power increases with later truncation times.

**Conclusions:**

This paper demonstrates the role of RMST estimators for survival analysis in the presence of main treatment effects and treatment-by-covariate interactions — highlighting the utility of RMST estimators in the analysis of trials with combined treatment effects. This paper offers further practical guidance on the strengths and limitations of parametric and non-parametric RMST estimators in the presence of model misspecification. It also serves as a case study for trialists wishing to explore RMST estimators by simulations tailored to their own research context.

**Supplementary Information:**

The online version contains supplementary material available at 10.1186/s13063-026-09666-8.

## Introduction

For many disease types, including cancers, randomised controlled trials (RCTs) that consider overall survival as the primary endpoint are widely accepted as the gold standard for testing a new experimental treatment against control in Phase III trials. Traditionally, the log-rank statistic has been utilised to estimate the hazard ratio for time-to-event (TTE) outcomes due to its interpretability and distributional results. As trials evolved, with a new emphasis on minimising patient risk and maximising patient benefit, Cox proportional hazards models became the new standard for the analysis of TTE outcomes. This semi-parametric method allows for greater flexibility and inclusion of covariates [1]. Generally, covariate adjustment leads to higher power and fewer required patients for Phase III trials [2].

### The role of restricted mean survival time for evaluation of treatment effects

In recent years, there has been increasing concern regarding the proportional hazards assumption. An imbalance in patient covariates after randomisation may challenge the interpretation of the hazard rate, which is determined for two patient groups with differing baseline characteristics [3]. The US Food and Drug Administration has published guidelines concerning inclusion of baseline covariates in TTE studies [4]. Trialists are increasingly turning to the restricted mean survival time (RMST) as an alternative measure to the hazard ratio [5]. As introduced by Royston et al. [6], the RMST evaluates the area under the survival curve up to selected truncation time $t^*$. The difference in RMST between treatment arms can be used as an estimator of treatment effect. In contrast to the usual Cox proportional hazards model, which estimates the survival distribution on each treatment arm at a single point in time, the RMST test statistic takes into account information accumulated over the survival function up to time $t^*$. For example, clinical trial data for which the estimated survival curves cross is often indicative of a treatment that only has a short-term benefit. For example, a surgical intervention may improve survival prospects for 6 weeks, yet over the course of 5 years, the non-surgical control treatment may prove to be more effective. RMST estimators may be particularly desirable when it is known or assumed that the proportional hazards assumption does not hold. Increasingly recognised as a robust and intuitive alternative to hazard ratios [7, 8], RMST is gaining increasing traction to estimate treatment effects both within meta-analyses [9, 10] and for survival analysis of Phase III trials [11, 12].

Our interest in RMST is motivated by trials that include multiple sources of information about treatment effect. This occurs both in trials assessing treatment effects across multiple endpoints [13] and in causal inference problems where the treatment affects the outcome through various pathways [14]. The traditional Cox proportional hazard approach can *only* evaluate multiple treatment TTE outcomes when the proportional hazard assumption is valid. However non-parametric and parametric RMST estimators may combine a multivariate vector of parameter estimates explaining treatment efficacy into a single univariate test statistic even when the proportional hazard assumption is violated.

Interaction terms can capture effect modification and potential time-varying treatment effects under non-proportional hazards.When one fits a model with a treatment and additional treatment-by-covariate interaction term, two parameters quantify treatment effect. We refer to an estimate of the combined effect of the treatment main effect and interaction terms as the combined treatment effect throughout this manuscript. In this paper, we evaluate the robustness of non-parametric and parametric RMST-based test statistics for assessing the combined contribution of the treatment main effect and its interaction with baseline covariates within a single hypothesis test for a randomised controlled trial.

RMST can be estimated in both a non-parametric and parametric manner. Whilst the type I error and power of RMST-based hypothesis tests have previously been assessed in crossing and non-crossing survival curve scenarios, the strengths and limitations of parametric RMST estimators have so far not been evaluated [15, 16]. Though the parametric RMST estimator is not usually utilised to assess treatment effect, it may provide trialists with more flexibility to investigate specific covariates and to use their knowledge to identify the anticipated knot-points for crossing curve scenarios — a particular limitation of the non-parametric RMST estimator [16].

For completeness, we assess the power of each parametric and non-parametric RMST estimator in both proportional hazard and non-proportional hazard scenarios.

For sensitivity analyses, we evaluate the RMST estimator in misspecified models. In a proportional hazards scenario, we omit explanatory covariates within a working model. We also consider a non-proportional hazard scenario, further evaluating performance when the estimated knot point model to assess treatment effect is misspecified [17]. Both types of misspecification are investigated as they induce bias and lead to a violation of the proportional hazard assumption [18].

The RMST estimator with a correctly specified model is used in the simulations as a benchmark against which this and other estimators are compared to in a variety of scenarios. This comparison is based on power and type I error rate. The choice of truncation time $t^*$ is also studied.

### RMST estimators in simulations

Simulation studies can support the analysis of power and type I error for different RMST-based hypothesis tests in the presence of various proportional hazard violations. In this paper, simulation studies are based on a two-arm randomized controlled trial comparing active Gamma interferon treatment with placebo for chronic granulomatous disease. In the ‘Statistical theory’ section, we give details of RMST estimation using non-parametric and parametric methods and the formulation of associated hypothesis tests. In the ‘Simulation study plan’ section, we present the simulation study plan and case study utilised to assess the robustness of RMST estimators. The ‘Estimators’ section details the specific estimators to be evaluated under both model misspecifications within this case study. Finally, the ‘Results’ section and the final ‘Discussion’ section are presented.

This paper provides practical guidance on the strengths and limitations of both parametric and non-parametric RMST estimators for combined treatment effects in the presence of proportional and non-proportional hazards scenarios. As well, this paper serves as a case study for trialists seeking to apply RMST methods within their own research context. Adhering to best practice recommendations [19], this paper presents a comprehensive methodological framework for the evaluation of treatment effects with RMST estimators using simulation studies — supporting trialists to critically assess the performance of RMST estimators under various assumption violations.

## Statistical theory

### Restricted mean survival time (RMST)

As presented by Royston and Parmar [6], the RMST is defined as the area below a survival curve *S*(*t*). This describes the probability that a patient survives up until at least time *t*, where $S(t)=\mathbb {P}(T>t)$ and *T* denotes time to some event of interest. The survival curve can be used as an outcome measure for time-to-event clinical trials. For some truncation point $t^*>0$, the RMST $\mu (t^*)$ is defined as,

1 $$\begin{aligned} \mu (t^*)=\int _0^{t^*} S(t) \; dt. \end{aligned}$$

Intuitively, $\mu (t^*)$ represents expected life expectancy until truncation time $t^*$ [6]. We consider the RMST as an estimand to measure treatment effect. We define the treatment effect as the difference in RMST at time $t^*$ between the treatment and control group and we test the null hypothesis that this difference is zero using the estimated difference and its variance. For two treatment arms with associated survival functions $S_1(t) \text { and } S_2(t)$, the treatment effect is defined as $\Delta (t^*) = (\mu _2-\mu _1) (t^*)$.

To evaluate the implications of identifying treatment efficacy with RMST, we perform a one-sided hypothesis test (at the 2.5% level) on the difference in treatment effect for three methods — estimating RMST using a non-parametric approach and RMST using a misspecified or correctly specified parametric approach, as detailed below.

We test the hypothesis:

2 $$\begin{aligned} \begin{array}{l} \text {H}_0: \Delta (t^*) =0, \\ \text {H}_1: \Delta (t^*)>0. \end{array} \end{aligned}$$

We test this hypothesis using an estimate of $\Delta (t^*)$ and an estimate of its sampling variance in the *Z*-test statistic. These are then compared to standard normal quantiles. We estimate $\mu _1$ and $\mu _2$ (with their corresponding standard errors) and then obtain the estimate and standard error of $\Delta$ using the independence of the treatment and control groups. For ease of notation, we subsequently write results in terms of $\hat{\Delta }(t^*)$, the estimated treatment effect, and $\text {s.e.}(\hat{\Delta }(t^*))$, its estimated standard error.

The *Z*-values are defined as,

3 $$\begin{aligned} Z = \frac{\hat{\Delta }(t^*)}{\text {s.e.}(\hat{\Delta }(t^*))}. \end{aligned}$$

A description of how $\hat{\Delta }(t^*)$ and $\text {s.e.}(\hat{\Delta }(t^*))$ are calculated is presented in the ‘Kaplan-Meier RMST estimator’ and ‘Cox-exponential RMST estimators’ sections.

Although in this manuscript we utilise a one-sided hypothesis designed to assess improvement in RMST, trialists may wish to utilise a two-sided hypothesis test if they would like to assess improvement and worsening in RMST within their trial. Trialists should also consider the sample size of their trial to ensure corresponding *Z*-values are well approximated by the normal distribution required for the validity of this test.

### Kaplan-Meier RMST estimator

We evaluate the area under the non-parametric Kaplan-Meier estimate of survival $\hat{S}(t)$. The Kaplan-Meier estimate of survival at time *t* is defined as,

4 $$\begin{aligned} \hat{S}(t) = \prod _{\tau _i<t}\left( 1-\frac{d(\tau _i)}{n(\tau _i)}\right) , \end{aligned}$$

where $d(\tau _i)$ is the number of patients who experience an event and $n(\tau _i)$ is the total number of patients in the study at time $\tau _i$.

When *N*, the number of time points where at least one event occurs, and $\tau _i \in \{\tau _1,...,\tau _N\}$ is such that $\tau _N \le t^* < \tau _{N+1}$, the non-parametric RMST estimator at time $t^*$ is defined as

5 $$\begin{aligned} \hat{\mu }(t^*) = \left( \sum \limits _{\tau _i \le t^*} \hat{S}(\tau _i)(\tau _i - \tau _{i-1}) \right) + \hat{S}(\tau _N) (t^* - \tau _N), \end{aligned}$$

where $\tau _0=0$. Note that calculating non-parametric RMST estimator relies on no model assumptions.

### Cox-exponential RMST estimators

We consider parametric RMST estimators under both proportional and non-proportional hazards scenarios. In this section, we focus on the more general setting in which the survival curves are permitted to intersect. Specifically, we assume a piecewise exponential model with a pre-specified time point $t_1$ at which the treatment covariate changes. The hazard functions for $t<t_1$ and $t \ge t_1$ follow distinct proportional hazard models. The time point $t_1$ thus acts as a knot point in the piecewise exponential model for survival data [20], allowing the treatment effect to vary across two time intervals. In this work, non-proportional hazards arise because the knot point $t_1$ is specified within the study time horizon. As the hazard model changes at $t_1$, the treatment effect differs for $t<t_1$ and $t \ge t_1$. If the corresponding knot point fell outside the period of interest, the hazards would be effectively proportional over the observed window and would not constitute an example of non-proportional hazards for our purposes.

For a piecewise exponential Cox model [20] with knot point $t_1$, the hazard associated with survival is characterised by baseline hazard functions $\lambda _{a,0}(t)$ and $\lambda _{0}(t)$ (which can vary across time), and components describing the way covariates effect the hazard, $\exp (\boldsymbol{\beta _a}^{T} {\textbf {X}})$ and $\exp (\boldsymbol{\beta }^{T} {\textbf {X}})$. The values of these components vary before and after the knot point respectively. Under the scenario of proportional hazards, we set $t_1=0$ and do not estimate $\lambda _{a,0} \text { or } \boldsymbol{\beta }_a$.

The hazard for a Cox proportional hazard model is given by,

6 $$\begin{aligned} h(t|\textbf{X})= \lambda _{a,0}(t)\exp (\boldsymbol{\beta }_a^T \textbf{X})1\!\!1\{t<t_1\} + \lambda _{0}(t)\exp (\boldsymbol{\beta }^T \textbf{X})1\!\!1\{t>t_1\}. \end{aligned}$$

Under proportional hazards, Eq. (6) simplifies to a cox-proportional hazards model. For simplicity, we assume that the baseline hazards $\lambda_{a,0}(t)$ and $\lambda_{0}(t)$ are constant. The cumulative hazard function $H(t|{\textbf {X}})$ describes the hazard accumulated from the beginning of the trial to time *t* and is defined as,

7 $$\begin{aligned} H(t|\textbf{X}) = \int _0^t h(u|{\textbf {X}}) \; du = \left\{ \begin{array}{ll} \lambda _{a} t \exp (\boldsymbol{\beta }_a^T \textbf{X}) & \text {if } t < t_1, \\ \lambda _{a} t_1 \exp (\boldsymbol{\beta }_a^T \textbf{X}) + \lambda (t-t_1) \exp (\boldsymbol{\beta }^T \textbf{X}) & \text {otherwise.} \end{array}\right. \end{aligned}$$

The survival function *S*(*t*) describing the probability that a patient survives up until at least time *t*, has the following relationship with the cumulative hazard,

8 $$\begin{aligned} \mathbb {P}(T>t) = S(t|{\textbf {X}}) = \exp (-H(t|{\textbf {X}})). \end{aligned}$$

The correctly specified RMST with all relevant covariates ${\textbf {X}}$ for some parameter vector $\boldsymbol{\beta }$ is given by:

9 $$\begin{aligned} \mu (t^*|\lambda _a,\lambda ,\boldsymbol{\beta }_a,\boldsymbol{\beta },\textbf{X})= & \int _0^{t^*} S(t) \; dt \end{aligned}$$

10 $$\begin{aligned}= & \left\{ \begin{array}{ll} \frac{(1-\exp \{-\lambda _a t^* \exp (\boldsymbol{\beta }_a^T \textbf{X})\})}{\lambda _a\exp (\boldsymbol{\beta }_a^T \textbf{X})} & \text {if } t^* < t_1 \\ \frac{(1-\exp \{-\lambda t^* \exp (\boldsymbol{\beta }^T \textbf{X})\})\exp \{\lambda t_1 \exp (\boldsymbol{\beta }^T \textbf{X})-\lambda _a t_1 \exp (\boldsymbol{\beta }_a^T \textbf{X})\}}{\lambda \exp (\boldsymbol{\beta }^T \textbf{X})} & \text {otherwise}. \end{array}\right. \end{aligned}$$

and the difference in RMST between treatment arms is $\Delta (t^*)$. A derivation of Eq. (10) is provided in Section 1 of the Supplementary Materials. In the context of non-proportional hazards, we wish to assess the early treatment effect for $t<t_1$.

For each parametric and non-parametric RMST estimator $\hat{\Delta }(t^*)$, we confirm that the *Z* test statistic associated with combined treatment effects are normally distributed by inspecting the histogram and Q-Q plots. These histograms and Q-Q plots for $n_\text {sim}=10^4$ simulations of *Z*-values under each RMST estimator are presented in Section 2 of the Supplementary Materials.

## Simulation study plan

### Aims

The aim of this study is to evaluate the robustness of both parametric and non-parametric RMST estimators under two forms of model misspecification:

1. Proportional hazards scenario — an explanatory variable is omitted from the fitted model.
2. Non-proportional hazards scenario — the knot point for the piecewise exponential model is misspecified.

### Working model

To evaluate the power of the RMST estimator under model misspecification, we run a simulation study where data is simulated from an exponential survival distribution which assumes proportionality of hazards.

The ‘cgd’ dataset within the ‘survival’ package [21] in R [22] is used as a case study. The ‘cgd’ dataset reports results of a placebo-controlled randomised trial of Gamma interferon for the treatment of chronic granulomatous disease. It evaluates time to serious infection observed in a sample of 128 patients. As each patient may have multiple infections within the observation window, we only consider the time to first serious infection for each patient. Patient survival times are generated using five covariates — some scalar baseline hazard $\lambda$ and four explanatory binary variables: ‘treatment’ (treatment or control) ‘inherit’ (pattern of inheritance), ‘sex’ (male or female), and a ‘treatment:inherit’ interaction. Let $\textbf{X}=(X_\text {Treatment},X_\text {Inherit},X_\text {Sex},X_\text {Treatment:Inherit})^T$ be a $4\times 1$ vector of covariates for a particular patient. The factor levels of each variable are presented in Table 1.

**Table 1:** Variable factor levels for the ‘cgd’ dataset

Variable	Factor level	Level value
$X_{\text {Treatment}}$	Control	0
	Gamma interferon	1
$X_{\text {Inherit}}$	X-linked	0
	Autosomal	1
$X_{\text {Sex}}$	Male	0
	Female	1

The true covariate coefficients are estimated using the original ‘cgd’ dataset and the ‘eha’ package in R with parameter values shown in Table 2 [23]. To evaluate RMST estimators under the first model misspecification, we set $\beta _3=0$ in the fitted model, while retaining its non-zero value in the data-generating mechanism. This induces omitted-variable bias and allows assessment of estimator robustness to covariate misspecification. Note that the true coefficient of ‘sex’ is also altered from − 0.402 to − 1 to emphasise the effect of model misspecification on the RMST estimators.

**Table 2:** Variables and coefficient values used to predict time to serious infection under proportional hazards as informed by the ‘cgd’ dataset

Variable	Coefficient value	Explanation
$\lambda : \text { Base hazard}$	0.0158	Base hazard
$\beta _1: \text { Treatment}$	− 1.117	Received treatment
$\beta _2: \text { Inherit}$	0.094	Autosomal
$\beta _3: \text { Sex}$	− 0.402	Female
$\beta _{12}: \text { Treatment:Inherit}$	0.475	Treatment:Autosomal

In general $\boldsymbol{\beta }_a$ and $\boldsymbol{\beta }$ may differ in several or even all its elements. To induce a scenario with non-proportional hazards, $\boldsymbol{\beta }_a$ and $\boldsymbol{\beta }$ are the covariate coefficients before and after knot point $t_1.$ The coefficient vectors, $\boldsymbol{\beta }_a=(\beta _{1,a},\beta _{2,a},\beta _{3,a},\beta _{12,a})^T$ and $\boldsymbol{\beta }=(\beta _{1},\beta _{2},\beta _{3},\beta _{12})^T,$ are each of the same dimension as $\textbf{X}$. The true knot point of the piecewise model is fixed throughout as $t_1=40$ and the treatment effects before and after $t_1$, given by $\beta _{1,a},\beta _{1}$, are altered and exaggerated to ensure that the survival curves cross. $\boldsymbol{\beta }_a$ and $\boldsymbol{\beta }$ differ only in the coefficient for the treatment effect ($\beta _{1,a} = -1.117$ and $\beta _1 = 0.750$) and the coefficient for Sex is set to its ‘true’ value − 0.402 in both intervals. We emphasise that fixing $t_1=40$ provides a benchmark for evaluating sensitivity to misspecification. In practice, the true knot point is unknown and some degree of misspecification is expected.

### Data generating mechanism

We generate the survival times *T* using the inverse-transform relationship [24],

11 $$\begin{aligned} T=\frac{-\log (U)}{h(t|{\textbf {X}})}, \end{aligned}$$

where $U \sim \text {Uniform}[0,1]$ and accordingly, $-\log (U)\sim \text {Exp}(1)$. Data is simulated in accordance with a Cox piecewise exponential model with a single knot point. For each of the model misspecifications, parameter values $\boldsymbol{{\beta }}$ used to generate survival times are presented in Table 2.

All regression coefficients are fixed across simulations. To provide a balanced design and avoid variability driven by unequal covariate prevalence, treatment, sex and inheritance pattern covariates are generated with 50% prevalence using independent Bernoulli(0.5) random variables. Censoring is simulated using an exponential random variable with rate 0.001, ensuring that approximately 10% of patients are randomly censored. The arrival window for patients to the study is 0–20 weeks and the study is concluded at 120 weeks so that maximum follow-up is 120 weeks. With these parameter values, overall censoring is approximately 40%.

Under both the proportional and non-proportional hazard scenarios, the trial sample size is fixed using simulation to ensure power under the correctly specified RMST estimator is approximately 80%. The total trial sample sizes for the proportional hazards and non-proportional hazards scenarios are 100 and 130, respectively.

**Fig. 1:**
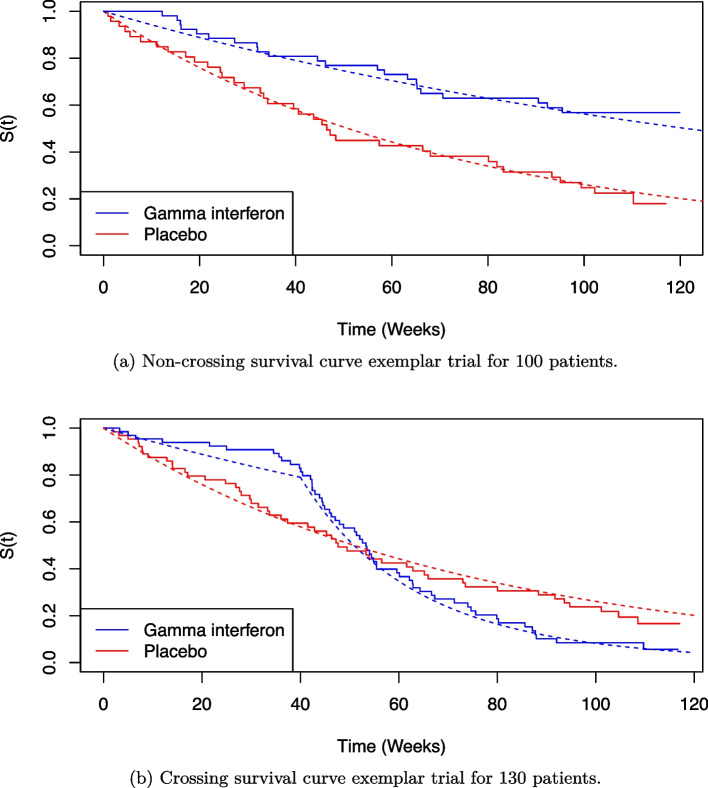
Kaplan-Meier plots under proportional hazards and non-proportional hazards scenarios for patients on control or Gamma interferon treatment. Dashed lines show the parametric value of the survival function for the parameter values of Table 2 for subfigures **a** and **b** accordingly

Figure 1 presents Kaplan-Meier plots for an example trial replication for both scenarios. The dashed lines represent the true survival function $S(t)$ averaged over covariate distributions for treatment, Gamma interferon, and control. In this data generating scheme, under non-proportional hazards, the experimental treatment is effective in the short-term but less effective than control in the long term. In Fig. 1, although the knot point occurs at 40 weeks, there is a delay before the curves cross at roughly 55 weeks. Hence, as $t^*$ increases, the RMST estimator is positive and increasing up to $t^*=55$. The area between survival curves reaches net zero at roughly 90 weeks and hence as $t^*$ increases, the RMST estimator is positive and decreasing between $t^*=55$ and $t^*=90$, and is negative and decreasing beyond $t^* = 90.$

### Performance measures

We evaluate the empirical bias and sampling variance of each estimator and the power and type I error rate of the *Z*-test based on it. The number of replications in the simulation study $n_\text {sim}$ is chosen to be $n_\text {sim} = 10^4$ to ensure that the Monte Carlo standard error for type I error estimates is maintained at 0.16%.

Bias is calculated with respect to the RMST estimand $\Delta(t^*)$, calculated as per Eq. 12 with true coefficient values as detailed in Table 2. Bias and the standard error (MCSE) of estimate are calculated using Morris et al. [19], where:

12 $$\begin{aligned} \text {bias}(\hat{\Delta}(t^*) ) = n_\text {sim}^{-1} \sum \limits _{i=1}^{n_\text {sim}} \hat{\Delta }(t^*)_i - \Delta(t^*) , \end{aligned}$$

13 $$\begin{aligned} \text {MCSE[bias(}\hat{\Delta }(t^*))] = \sqrt{\frac{1}{n_\text {sim}(n_\text {sim}-1)} \sum \limits _{i=1}^{n_\text {sim}}(\hat{\Delta }(t^*)_i - \bar{\Delta }(t^*))^2}. \end{aligned}$$

## Estimators

We assess the robustness of non-parametric and misspecified parametric RMST estimators under a proportional hazards example where one covariate ’sex’ is set to zero. Whilst the fully parametrised RMST estimator usually cannot be obtained in practice, it provides an upper bound to assess the robustness of the non-parametric and misspecified parametrically defined RMST estimators.

Following the structure of the case study described in ‘Simulation study plan’ section, we consider the explanatory variables $\textbf{X}=(X_\text {Treatment},X_\text {Inherit},X_\text {Sex},X_\text {Treatment:Inherit})^T$, where treatment, sex and inheritance pattern are generated independently from Bernoulli (0.5) distributions.

Non-parametric RMST estimators are evaluated using the ‘rmst2’ function in the ‘survRM2’ package [25] in R. The variance of the non-parametric RMST estimator is estimated using the ‘survRM2’ function which uses the Greenwood’s plug-in estimator for asymptotic variance.

We derive the area under a Cox-exponential survival curve similarly to the ‘Statistical theory’ section, given estimated parameter values $\boldsymbol{\hat{\theta }}=(\theta _1, \theta _2, \theta _3, \theta _{12})^T$ and $\hat{\lambda }_1$,

14 $$\begin{aligned} \hat{\mu }_\text {full}(t^*|\boldsymbol{\hat{\theta }}, \hat{\lambda }_1,\textbf{X}) = \frac{(1-\exp (-\hat{\lambda }_1 t^* \exp (\textbf{X}^T \boldsymbol{\hat{\theta }})))}{\hat{\lambda }_1 \exp (\textbf{X}^T \boldsymbol{\hat{\theta }})}. \end{aligned}$$

The treatment effect estimator is defined as,

15 $$\begin{aligned} \hat{\Delta }_\text {full}(t^*|\boldsymbol{\hat{\theta }}, \hat{\lambda }_1) = \hat{\mu }_\text {full}(t^*|\hat{\boldsymbol{\theta }},\hat{\lambda }_1,X_\text {Treatment}=1)-\hat{\mu }_\text {full}(t^*|\boldsymbol{\hat{\theta }},\hat{\lambda }_1,X_\text {Treatment}=0). \end{aligned}$$

The RMST is estimated by maximum likelihood using ‘phreg’ function in R package ‘eha’ [23].

A full derivation of $\hat{\Delta }_\text {full}(t^*|\hat{\boldsymbol{\theta }}, \hat{\lambda }_1)$ under our simulation scenario is presented in Section 3 of the Supplementary Materials. We approximate the variance of the RMST estimator by the delta method. Details can be found in Section 4 of the Supplementary Materials.

Under the working model used to simulate outcomes, survival is dependent on the ‘sex’ covariate. To evaluate the effect of misspecification on the power of the parametric RMST estimator, we misspecify the estimator by setting the coefficient for sex to zero in estimating $\beta$.

Given parameter estimates $\boldsymbol{\hat{\gamma }=(\gamma _1, \gamma _2, \gamma _3, \gamma _{12})^T}$ and $\hat{\lambda }_2$, the misspecified parametric RMST estimator and RMST difference can be defined similarly to Eqs. 14 and 15.

We also investigate the robustness of the parametric and non-parametric RMST estimators under a non-proportional hazards scenario with a misspecified knot-point. That is, data is simulated using $t_1=40$ but estimation of the parametric RMST estimator is assessed under a range of misspecified knot points $\tilde{t}_1$ in place of $t_1$ in Eq. 14. Knot point $t_1$ is fixed and defined prior to commencement of the trial. Methods are available which allow data driven estimation of $t_1$ [26], however these methods are generally most appropriate for exploratory purposes and we have chosen to fix $t_1$ to ensure that parameter estimation is robust and type I error rates are protected.

## Results

To assess Aim 1 (robustness under proportional hazards scenario with omitted covariate), the power and type I error rate is presented as $t^*$ varies from 1 to 120 weeks with $\beta _3$ fixed at $-1$. We also evaluate these as $\beta _3$ varies from − 2 to 2 and $t^*$ is fixed at 100 weeks. To assess Aim 2 (robustness under non-proportional hazards with misspecified knot point), we evaluate the power and type I error rate as $t^*$ varies from 1 to 120 weeks with knot point $\tilde{t}_1$ misspecified at 50 weeks. We also evaluate these as $\tilde{t}_1$ varies from 15 to 40 weeks and $t^*$ is fixed at 40 weeks to evaluate early treatment effect.

Maximum likelihood estimation of RMST is defined only when one event is observed for every possible combination of patient covariates. In the proportional hazards scenario with a sample size of 100 patients, some simulated clinical trial realisations may contain no events for certain combinations of treatment, inheritance pattern, and sex. Replications in which this occurs are excluded from the analysis, the reported results are based on the number of successful replications remaining.

Non-parametric RMST estimator is well defined only when at least one patient remains at risk at the time point $t^*$. As $t^*$ increases, more trials are excluded from the non-parametric power calculations. Only 1 trial was excluded from power calculations at 107 weeks. By 114 weeks, 113 of trials could not be used as part of power calculations. As $\beta _3$ becomes more positive, and more events are observed, patients are less likely to remain on the trial until 120 weeks. When $\beta _3$ is set at 0.1, 5 trials cannot be used for power calculations. This increases steadily until $\beta _3$ is set to 2, where 115 trials cannot be used to evaluate power out of 10,000 trial replications. 

Although parametric RMST estimators do not share this limitation (since the assumed model permits extrapolation beyond the observed follow-up) we did not calculate their power in scenarios where power could not be computed for the non-parametric estimator. This restriction was imposed to maintain a fair and unbiased comparison across methods.

### Misspecification by neglecting explanatory covariate

**Fig. 2:**
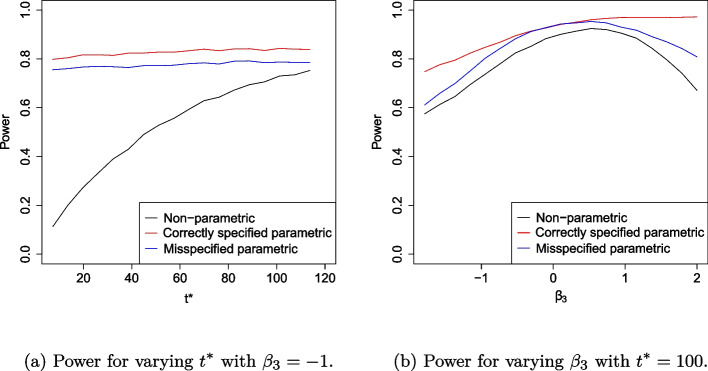
Simulation study results showing power when data is fit to a Cox proportional hazards model for proportional hazards scenario when the model is misspecified by omitting explanatory covariate

**Fig. 3:**
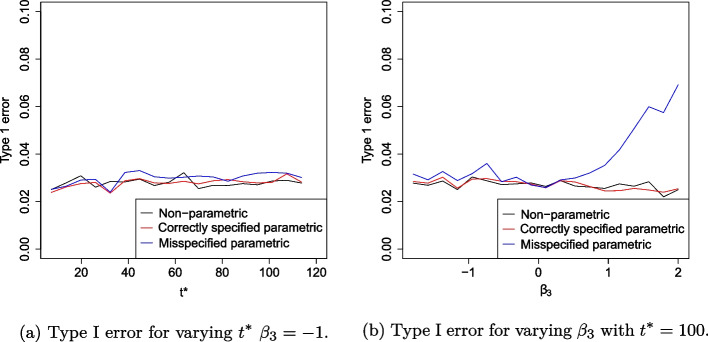
Simulation study results showing type I error when data is fit to a Cox proportional hazards model for proportional hazards scenario when model is misspecified by omitting explanatory covariate

The power and type I error rate as $t^*$ and $\beta _3$ varies is presented in Figs. 2 and 3. As anticipated, the power of the correctly specified parametric RMST estimator is always higher than the non-parametric and misspecified parametric estimator. As it follows the true working model, the correctly specified parametric RMST estimator provides an upper bound for performance.

Under the misspecified parametric model, power remains constant across different choices of $t^*$, unlike the non-parametric model’s power which is greater for larger values of $t^*$. For increasing $t^*$, the difference in the area under the curve for the treatment and control increases. For the non-parametric RMST estimator, it increases the magnitude of the combined treatment effect. The associated standard error increases but at a slower rate, thus increasing the magnitude of the *Z*-value and power. For both parametric RMST estimators, the power does not depend on $t^*$. The difference in the area under the survival curve and the standard error of the associated statistic increase proportionately, keeping the *Z*-value approximately constant.

As the magnitude of $\beta _3$ coefficient increases, both the non-parametric and the misspecified parametric estimator’s power decreases. The test based on the misspecified parametric RMST estimator achieves power comparable to the correctly specified model for small values of $\beta _3$. The correctly specified estimator always has the largest power as $\beta _3$ varies, as expected. When $t^*$ is fixed, the power of non-parametric RMST estimator decreases as the magnitude of $\beta _3$ increases as treatment survival curves become closer together and the difference in RMST estimator decreases. For the misspecified parametric RMST estimator, increasing the magnitude of the unaccounted $\beta _3$ parameter reduces power. In contrast, the power of the correctly specified parametric RMST estimator increases as $\beta _3$ becomes more positive. Whilst the size of the combined treatment effect decreases as the magnitude of $\beta _3$ increases, so too does the standard error of the *Z*-value. Incidence is more likely and its count increases at $t^*$ increases. The greater number of incidence events provides more information and hence reduces the standard error and increases the magnitude of the respective *Z*-value.

The type I error for the non-parametric RMST estimator and correctly specified parametric RMST estimator remains relatively stable to varying $t^*$ and $\beta _3$ values. As $t^*$ varies, the non-parametric, correctly specified parametric and misspecified parametric RMST estimators have an empirical mean type I error rate of 2.7, 2.8 and 3.0%, respectively.

However, as $\beta _3$ becomes more positive, the type I error rate for the misspecified model inflates above the expected 2.5% to 7.3%. For this estimator, the standard error of the estimator deflates as more events are observed. This increases the magnitude of some *Z*-values, resulting in more rejections of the null hypothesis and inflating the type I error rate.

All type I errors rates in these simulations exceed the expected 2.5% error rate. These results reflect published findings [27, 28] that type I error rates can be inflated due to small trial sample size (100 patients) as the sample size is too small for the asymptotic normality to apply. This increases the observed type I error rate by around 0.2%. Further simulations (not shown) show that for a sample size of 250 patients, all type I error rates are within the expected range according to a binomial distribution with $10^4$ trial simulations. A more precise characterisation of the finite-sample null distribution of the test statistic could be obtained using simulation-based methods, along with an assessment of whether it can be adequately approximated by a scaled chi-squared distribution. Such an approach may improve control of type I error rates in small-sample settings.

**Fig. 4:**
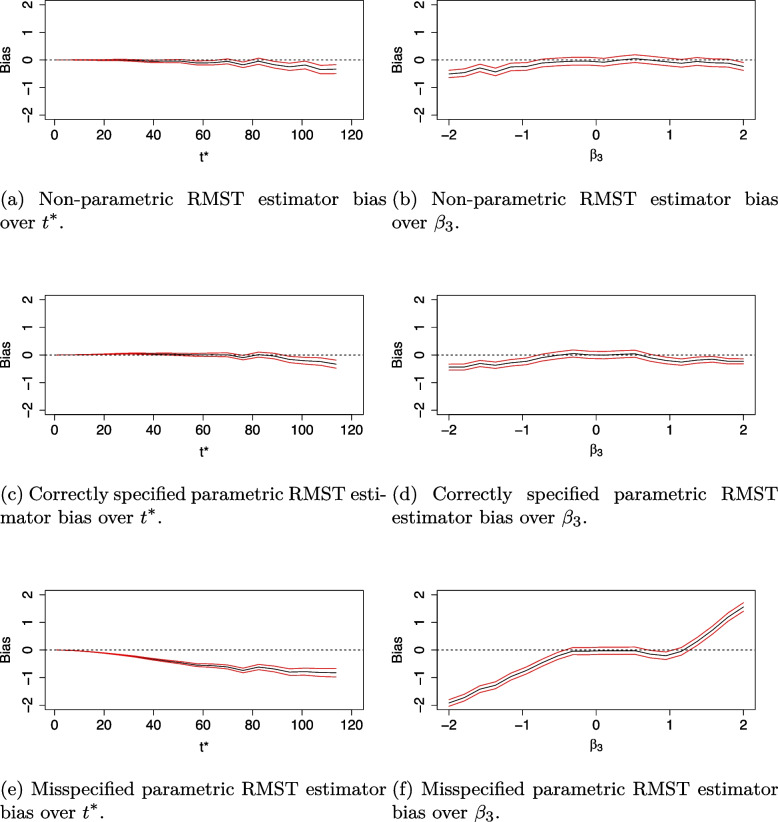
Bias of estimators under the proportional hazards scenario for alternative hypothesis (black line) with $\pm 2$*standard error of estimate in red

The bias of each RMST estimator with standard errors is presented in Fig. 4. The non-parametric RMST estimator has the smallest bias over the range of $t^*$ and $\beta _3$. Bias is also very small for the fully-specified parametric RMST estimator. The misspecified parametric RMST estimator appears substantially biased over varying $t^*$ and $\beta _3$. It underestimates treatment effect for varying $t^*$ and negative $\beta _3$ values, whilst it overestimates treatment effect for positive $\beta _3$ values.

Sections 4 and 5 of the Supplementary Materials contain tabulated and graphical mean summaries of *Z*-values, treatment effects, and associated standard error for power and type I error rate calculations as $t^*$ and $\beta _3$ varied.

### Misspecification of knot point for non-proportional hazard example

**Fig. 5:**
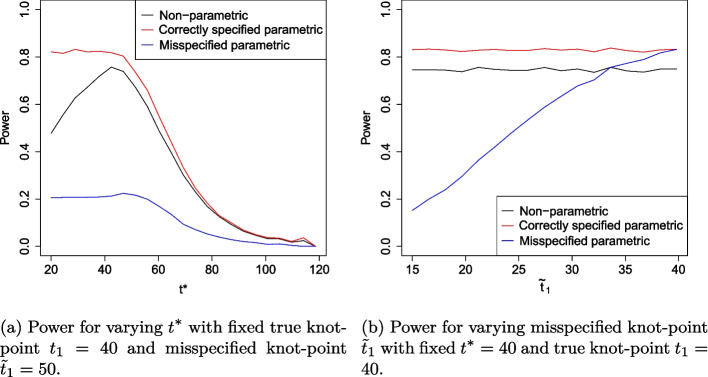
Simulation study results showing power when data is fit to a piecewise exponential Cox model with non-proportional hazards survival curves

**Fig. 6:**
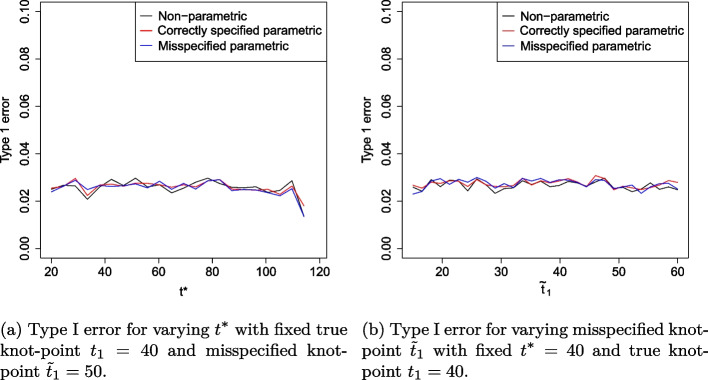
Simulation study results showing type I error rates when data is fit to a piecewise exponential Cox model with crossing non-proportional hazard survival curves

**Fig. 7:**
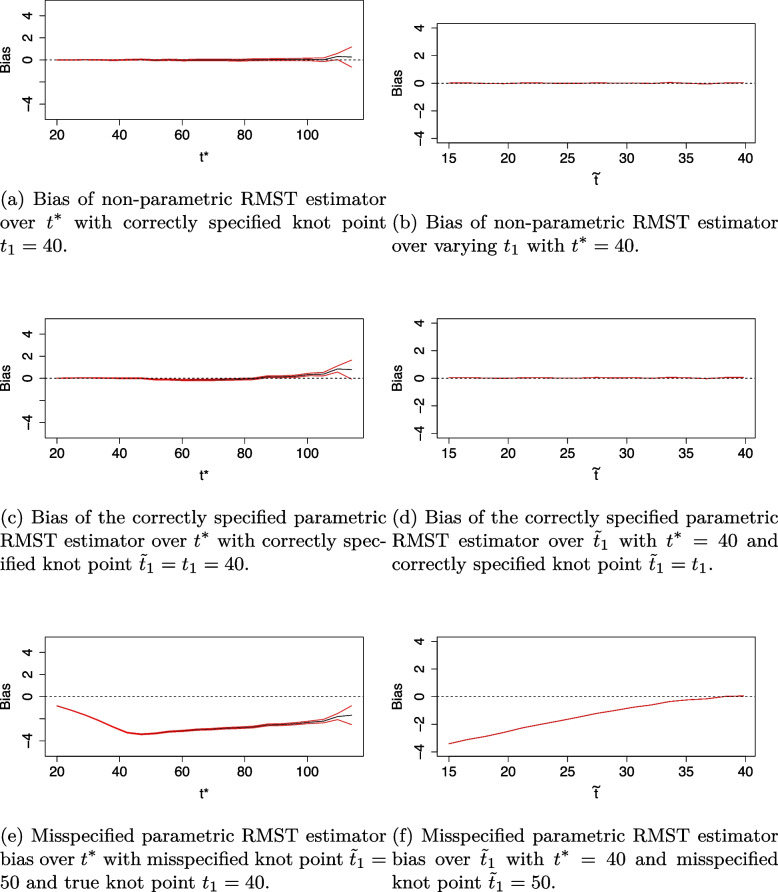
Bias of estimators under the non-proportional hazards scenario for alternative hypothesis (black line) with $\pm 2$*standard error of estimate in red when the true knot point $t_1=40$

Under the non-proportional hazard scenario, Fig. 5a shows the effect of varying $t^*$ on power when the knot point is misspecified at week 50. As expected, for all methods considered, as $t^*$ increases, power increases up to $t^*=50$ and decreases beyond $t^*=50.$ All methods appear highly sensitive to the choice of $t^*$, with power decreasing rapidly as $t^*$ increases beyond the time the survival curves cross. The correctly specified fully parametric estimator is most efficient in all cases, reaching power of approximately 80% compared to a maximum power of 75% for the non-parametric model. Figure 5b highlights the sensitivity of the parametric RMST estimator to knot point misspecification. The misspecified parametric RMST estimator increases in efficiency as the model specified knot-point, $\tilde{t}_1$, approaches its true value. An incorrect assumption that the treatment efficacy changes at 20 weeks reduces power to roughly 40% and an incorrect assumption that efficacy changes at 60 weeks reduces power to almost zero. In fact, the parametric model only yields a more efficient estimator than its non-parametric counterpart when $\tilde{t}_1$ is within 6 weeks of $t_1$. Figure 6a summarises the effect of varying $t^*$ on the type I error rates when a non-proportional hazards model is assumed. Type I error rate is protected at 2.5% for all methods. This is as expected since under $H_0$, the survival curves are identical and do not cross so this feature is not affected by changes in $t^*$. We note that there is more noise in Fig. 6a than in Fig. 3, this is because twice as many parameters are estimated due to the piecewise structure of the fitted model. Figure 6b shows the effect of model misspecification on type I error rates. We find that the assumed value of the knot-point at the model fitting stage does not affect type I error. This is because under $H_0$, there is no knot point. The bias of each RMST estimator with standard errors is presented in Fig. 7.

In Section 6 of the Supplementary Materials, we discuss the effects of varying $t^*$ when RMST is used to analyse a trial in which the control is initially more effective than the experimental treatment but then becomes less effective later and the survival curves intersect (such that the curves intersect in the opposite direction to what we have discussed here). To address this problem, we simply exchange the treatment labels in the simulation. We see that the type I error rates are unaffected but the power is again highly susceptible to changes in $t^*$.

## Discussion

### Misspecified covariate

For small model misspecifications, both parametric and non-parametric RMST estimators have satisfactory power. However trialists should be wary of the inflated type I error rate of misspecified RMST estimators under severe model misspecification. Non-parametric RMST estimators may be better suited for hypothesis tests when trialists suspect that some covariates are missing from analysis.

The power and type I error rate of *Z*-tests derived from misspecified models that utilise the RMST estimator to assess treatment efficacy is dependent on the magnitude of the coefficient associated with the covariate. In the context of the one-sided hypothesis test, the loss in power is greater as $\beta _3$ becomes more negative.

When the parametric model holds or misspecification is low, the non-parametric RMST estimator has lower power than its parametric counterpart, with power remaining reliant on time of analysis $t^*$. However, compared to a misspecified model which neglects an explanatory covariate, a non-parametric RMST estimator has substantially lower bias and type I error rate than the misspecified alternative as both $t^*$ and $\beta _3$ vary. When the type I error rate is evaluated as the unknown parameter varies in magnitude, the misspecified estimator’s type I error rate increases to 7.3% as $\beta _3$ becomes more positive. In contrast, the correctly specified and non-parametric estimator’s type I error rate remains unchanged (approximately 3%) as the size of $\beta _3$ varies. As $t^*$ varies, the non-parametric, misspecified and correctly specified estimators all have similar, stable type I error values.

### Misspecified knot point

If the survival functions for the two groups interesect, then the RMST estimator is useful for hypothesis testing under suitable choices of parameter values and reasonable prior knowledge of the survival distribution.

When the knot point $t_1$ is misspecified, the non-parametric RMST estimator consistently has better power compared to a misspecified parametric RMST estimator unless the misspecified knot point is close to the true knot point. However, it is important to note that the non-parametric RMST estimator cannot be evaluated for values of $t^*$ beyond a certain threshold. Therefore, the value of $t^*$ should be clinically meaningful and the timing of the trial analysis should be planned appropriately in accordance with the chosen value of $t^*.$

We see that type I error rates are protected at the 2.5% level for each method, even when the model is subject to misspecification. This is because under $H_0$, survival curves on the treatment and control are equal and the specification of the knot point is inconsequential.

As with many estimators, accurate estimation of the knot point $t_1$ is critical for obtaining reliable inference. Whilst we assume a fixed knot point throughout this paper, the choice of knot location is an important modelling consideration. Previous research has examined the performance of flexible parametric survival models under different knot specifications, often using information criteria such as the Akaike Information Criterion (AIC) and the Bayesian Information Criterion (BIC), alongside computationally more intensive approaches [6, 29]. However, we do not investigate knot selection in this manuscript.

### Extensions

Within this paper, we explore RMST estimators for under both proportional and non-proportional hazards. However, simulated survival times and parametric models used to estimate the RMST estimator are Cox-exponential proportional hazard models either side of knot point $t_1$, with the goal of presenting a best case scenario for power and type I error rate calculations. For the non-proportional hazard scenario where treatment arm survival curves are non-parallel, we suppose survival curves converge to knot point $t_1$. Future work could consider the scenario where survival curves diverge, or where there are regression parameters specific to the two arms of the trial.

When power is evaluated as $\beta _3$ varies, $t^*$ is chosen to be 100 weeks as previous literature has suggested that $t^*$ should be close to the last observed survival time [6]. New literature has presented the window mean survival time (WMST) [30] which evaluates the mean survival time between an upper and lower time horizon, $t^*_0$ and $t^*_1$, respectively. The WMST is a generalisation of the RMST estimator with $t^*_0=0$ and $t^*_1=t^*$. A further extension to this work could consider power and type I error comparisons for the non-parametric and parametric WMST methods. In this paper, we utilise the RMST difference due to its popularity and common usage as an estimand in RMST literature. An alternate RMST estimand is the RMST ratio [31], evaluating average survival between groups over a fixed period. Estimates of RMST ratio can be obtained similarly from the same RMST estimators. Future work could evaluate the performance of the RMST ratio, though we anticipate comparative performance to RMST difference estimators.

This paper only evaluates the RMST in a fixed sample size trial design. Patients are enrolled to the study during a fixed accrual period and analysis is completed at a fixed time. The trial sample size was fixed to attain 80% power for the fully specified RMST estimator with a sample size of 100 and 130 patients under proportional and non-proportional hazard scenarios respectively. Future work could further explore performance across varying sample sizes.

This manuscript considers the role of RMST to estimate combined treatment effects for RCTs with treatment and baseline covariate interactions. However, the proposed RMST framework may further extend to factorial designs to assess treatment effects in which multiple treatments are randomised and their interactions are incorporated within the model. Further, this work could be extended to evaluate the RMST estimator within an adaptive trial setting where efficacy is evaluated during interim-analysis time points. This could include the evaluation of power and the type I error rate for the RMST estimator within a group sequential trial design.

## Conclusion

Whilst parametric RMST estimators can offer greater flexibility than their non-parametric counterparts, they are generally only appropriate for estimating combined treatment effects when any model misspecifications are minimal. In contrast, non-parametric RMST estimators may be preferable in situations when larger model misspecifications are likely, with robustness to misspecification ensuring type I error rates and power are stable under such circumstances.

As scientific interest and utilisation of RMST estimators continue to grow in the clinical trial field, we hope this paper highlights the expanding opportunities for RMST to be applied in practice for estimating combined treatment effects.

## Supplementary Information

Supplementary Material 1.

## Data Availability

The code associated with this paper is available in the rmst-simulations repository, https://github.com/alemily100/rmst-simulations.

## Abbreviations

TTE: Time-to-event
RCT: Randomised controlled trial
RMST: Restricted mean survival time
