# Supplementary material for: Estimation of combined treatment effects by restricted mean survival time
Source: Trials. 2026 Apr 1;27:364. doi: 10.1186/s13063-026-09666-8 (PMC13169728; doi:10.1186/s13063-026-09666-8)
Supplement: Supplementary file 1 — Supplementary Material 1. [file 13063_2026_9666_MOESM1_ESM.pdf]

Supplementary materials

1 Deriving RMST for crossing curves

For $t^* \geq t_1$,

$$\mu(t^*|\lambda_a,\lambda,\boldsymbol{\beta}_a,\boldsymbol{\beta},\mathbf{X}) = \int_0^{t^*} S(t)\; dt \tag{1}$$

$$= \int_0^{t^*} \frac{\exp\{-\lambda_a t_1 \exp(\beta_a^T \mathbf{X}) + \lambda t_1 \exp(\beta^T \mathbf{X})\}}{\exp(\lambda t \exp(\beta^T \mathbf{X}))}\; dt \tag{2}$$

$$= \exp\{-\lambda_a t_1 \exp(\beta_a^T \mathbf{X}) + \lambda t_1 \exp(\beta^T \mathbf{X})\} \int_0^{t^*} \exp(-\lambda t \exp(\beta^T \mathbf{X}))\; dt \tag{3}$$

$$= \exp\{-\lambda_a t_1 \exp(\beta_a^T \mathbf{X}) + \lambda t_1 \exp(\beta^T \mathbf{X})\} \left[\frac{-\exp(-\lambda t \exp(\beta^T \mathbf{X}))}{\lambda \exp(\beta^T \mathbf{X})} + \frac{1}{\lambda \exp(\beta^T \mathbf{X})}\right]_0^{t^*} \tag{4}$$

$$= \exp\{-\lambda_a t_1 \exp(\beta_a^T \mathbf{X}) + \lambda t_1 \exp(\beta^T \mathbf{X})\} \left[\frac{1 - \exp\{-\lambda t^* \exp(\beta^T \mathbf{X})\}}{\lambda \exp(\beta^T \mathbf{X})}\right] \tag{5}$$

2 Checking normality of Z-values

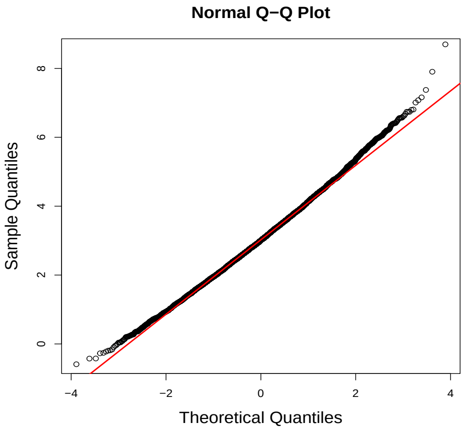

(a) Q-Q plot of Z-values.

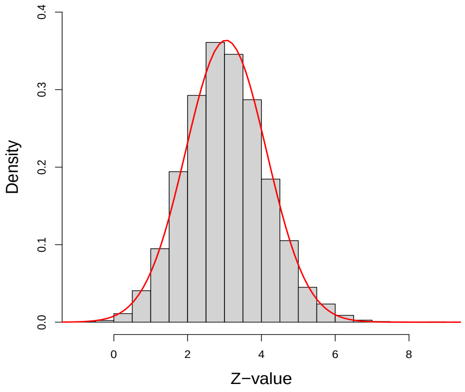

(b) Histogram of Z-values with overlayed normal distribution using sample mean and sample standard error.

Fig. S1: Normality assessment of the non-parametric RMST estimator for $1,000$ Z-values.

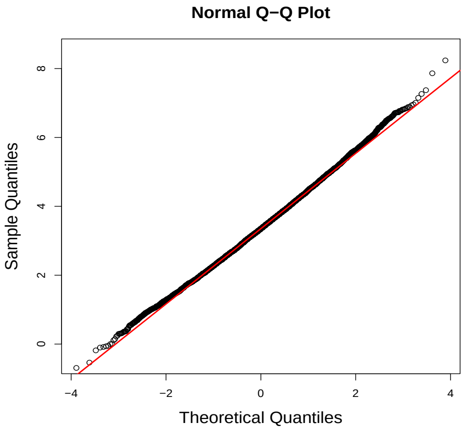

(a) Q-Q plot of Z-values.

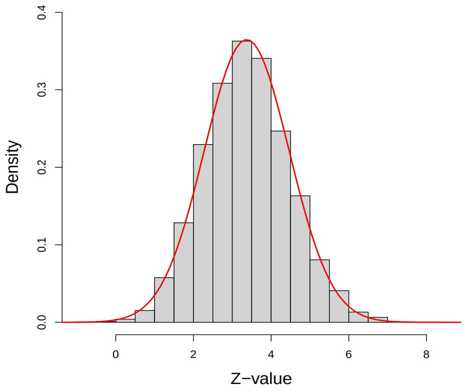

(b) Histogram of Z-values with overlayed normal distribution using sample mean and sample standard error.

Fig. S2: Normality assessment of the correctly specified parametric RMST estimator for $1,000$ Z-values.

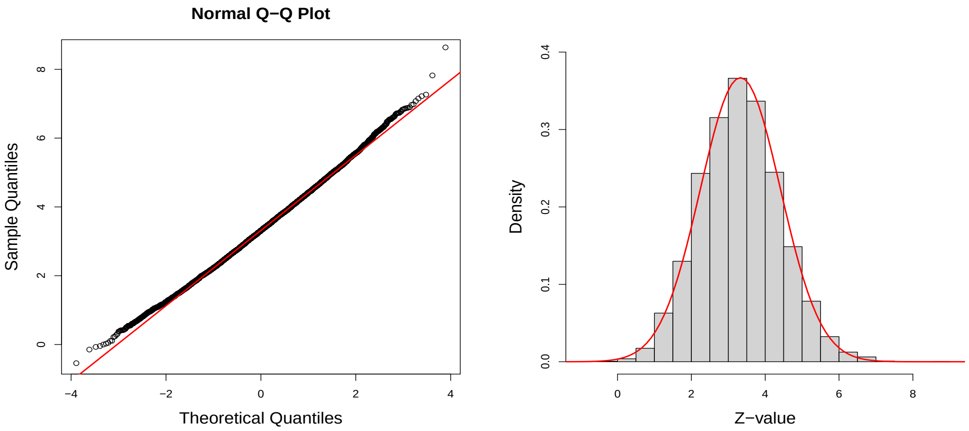

(a) Q-Q plot of Z-values.

(b) Histogram of Z-values with overlayed normal distribution using sample mean and sample standard error.

Fig. S3: Normality assessment of the misspecified parametric RMST estimator for $1,000$ Z-values.

3 Deriving the correctly specified RMST estimator

Within this case study, the covariates $X_{\text{Treatment}}$, X_{Inherit} and X_{sex} are generated using Bernoulli(0.5) random variables. In the following section we use the indicator function to show the presence of each covariate. For example, $\mathbb{1}_{X_{\text{Treatment}}} \in \{0, 1\}$ and is equal to 1 if a patient is on the treatment arm and 0 if the patient is on the control arm. The interaction between 'treatment' and 'inherit' is defined as $X_{\text{Treatment:Inherit}} = \min(X_{\text{Treatment}}, X_{\text{Inherit}})$. Table 1 in the main manuscript presents the associated values of each indicator function for all covariates.

3.1 Treatment arm

The instances, and probability of occurrence, for each possible combination of covariates for patients in the treatment arm is presented in Table S1.

Given $X_{\text{Treatment}} = 1$,

$\mathbb{1}_{X_{\text{Inherit}}}$	$\mathbb{1}_{X_{\text{Treatment:Inherit}}}$	$\mathbb{1}_{X_{\text{Sex}}}$	**Probability of occurrence**
1	1	1	0.25
1	1	0	0.25
0	0	1	0.25
0	0	0	0.25

Table S1: All combinations of covariate indicators for patients in the treatment arm, reported alongside probability of occurrence.

Hence, the associated corresponding survival function $S_1(t)$ for patients in the treatment arm with vector of fitted parameter coefficients $\hat{\boldsymbol{\theta}}$ and fitted base hazard $\hat{\lambda}_1$ is,

$$\hat{S}_1(t) = 0.25\exp(-\hat{\lambda}_1 t\exp(\hat{\theta}_1 + \hat{\theta}_2 + \hat{\theta}_{12} + \hat{\theta}_3)) + 0.25\exp(-\hat{\lambda}_1 t\exp(\hat{\theta}_1 + \hat{\theta}_2 + \hat{\theta}_{12}))+$$
$$+ 0.25\exp(-\hat{\lambda}_1 t\exp(\hat{\theta}_1 + \hat{\theta}_3)) + 0.25\exp(-\hat{\lambda}_1 t\exp(\hat{\theta}_1)).$$

3.2 Control arm

The instances, and probability of occurrence, for each possible combination of covariates for patients in the control arm is presented in Table S2.

Given $X_{\text{Treatment}} = 0$,

$\mathbb{1}_{X_{\text{Inherit}}}$	$\mathbb{1}_{X_{\text{Treatment:Inherit}}}$	$\mathbb{1}_{X_{\text{Sex}}}$	**Probability of occurrence**
1	0	1	0.25
1	0	0	0.25
0	0	1	0.25
0	0	0	0.25

Table S2: All combinations of covariate indicators for patients in the control arm, reported alongside probability of occurrence.

Similarly, the associated survival function under the control arm is,

$$\hat{S}_0(t) = 0.25\exp(-\hat{\lambda}_1 t\exp(\hat{\theta}_2+\hat{\theta}_3))+0.25\exp(-\hat{\lambda}_1 t\exp(\hat{\theta}_2))+0.25\exp(-\hat{\lambda}_1 t\exp(\hat{\theta}_3))+0.25\exp(-\hat{\lambda}_1 t).$$

In this instance,

$$\begin{aligned}\hat{\Delta}_{\text{full}}(t^*|\hat{\boldsymbol{\theta}},\hat{\lambda}_1) &= \hat{\mu}_{\text{full}}(t^*|\hat{\boldsymbol{\theta}},\hat{\lambda}_1, X_{\text{Treatment}}=1) - \hat{\mu}_{\text{full}}(t^*|\hat{\boldsymbol{\theta}},\hat{\lambda}_1, X_{\text{Treatment}}=0),\\ &= \int_0^{t^*}\hat{S}_1(t)\,dt - \int_0^{t^*}\hat{S}_0(t)\,dt.\end{aligned}$$

A similar approach is used to estimate the mis-specified parametric RMST.

4 Approximation of parametric RMST variance using the delta method

This method approximates the variance as,

$$\text{Var}((\mu_2 - \mu_1)(t^*)) \approx \nabla((\mu_2 - \mu_1)(t^*))^T \, \frac{\Sigma}{n} \, \nabla((\mu_2 - \mu_1)(t^*)). \tag{6}$$

where n is the number of covariates, Σ is the variance-covariance matrix for these variables, and,

$$\nabla((\hat{\mu}_2 - \hat{\mu}_1)(t^*)) = \left[\frac{\partial((\hat{\mu}_2 - \hat{\mu}_1)(t^*))}{\partial(\hat{\lambda})}, \frac{\partial((\hat{\mu}_2 - \hat{\mu}_1)(t^*))}{\partial(\hat{\beta}_1)}, \ldots, \frac{\partial((\hat{\mu}_2 - \hat{\mu}_1)(t^*))}{\partial(\hat{\beta}_n)} \right]^T . \tag{7}$$

5 Tabulated Z-value breakdown

Relevant to the Results section of the main manuscript, below we present a breakdown of mean standard error, treatment effect, and Z-values used to assess power and type I error as t^* and β_3 varied.

	Non-parametric estimator				Correctly specified parametric estimator				Misspecified parametric estimator			
t^*	Z	$\Delta(\hat{t}^*)$	s.e($\Delta(\hat{t}^*)$)	Power	Z	$\Delta(\hat{t}^*)$	s.e($\Delta(\hat{t}^*)$)	Power	Z	$\Delta(\hat{t}^*)$	s.e($\Delta(\hat{t}^*)$)	Power
**1.00**	0.44	0.00	0.01	0.00	2.63	0.00	0.00	0.81	2.54	0.00	0.00	0.76
**7.26**	0.83	0.16	0.19	0.12	2.66	0.17	0.06	0.81	2.55	0.14	0.06	0.77
**13.53**	1.12	0.54	0.47	0.21	2.71	0.55	0.20	0.82	2.58	0.48	0.19	0.77
**19.79**	1.34	1.11	0.82	0.28	2.75	1.14	0.41	0.83	2.61	1.00	0.38	0.78
**26.05**	1.53	1.85	1.21	0.34	2.79	1.90	0.68	0.83	2.63	1.68	0.64	0.78
**32.32**	1.68	2.73	1.63	0.39	2.82	2.80	0.99	0.83	2.66	2.51	0.94	0.79
**38.58**	1.80	3.72	2.08	0.45	2.83	3.81	1.34	0.83	2.66	3.45	1.29	0.78
**44.84**	1.93	4.87	2.53	0.49	2.87	4.95	1.72	0.84	2.68	4.52	1.68	0.78
**51.11**	2.04	6.11	3.01	0.54	2.88	6.17	2.14	0.84	2.70	5.69	2.10	0.79
**57.37**	2.13	7.37	3.49	0.57	2.91	7.48	2.57	0.84	2.71	6.93	2.55	0.79
**63.63**	2.22	8.78	3.98	0.61	2.94	8.89	3.03	0.84	2.74	8.31	3.03	0.79
**69.89**	2.32	10.30	4.48	0.63	2.96	10.35	3.51	0.85	2.76	9.73	3.53	0.80
**76.16**	2.37	11.69	4.98	0.66	2.96	11.77	4.00	0.84	2.75	11.13	4.06	0.80
**82.42**	2.46	13.39	5.48	0.69	3.01	13.45	4.5	0.85	2.80	12.82	4.59	0.80
**88.68**	2.51	14.87	5.98	0.70	3.02	15.01	5.00	0.85	2.81	14.36	5.13	0.80
**94.95**	2.56	16.43	6.48	0.72	3.01	16.52	5.52	0.85	2.80	15.88	5.70	0.80
**101.21**	2.63	18.17	6.98	0.74	3.02	18.14	6.05	0.86	2.82	17.56	6.27	0.80
**107.47**	2.66	19.69	7.48	0.76	3.03	19.80	6.58	0.85	2.82	19.22	6.85	0.81
**113.74**	2.71	21.41	7.98	0.76	3.03	21.41	7.11	0.85	2.83	20.92	7.43	0.80

Table S3: Mean simulated Z-value, treatment effect, and associated standard error to assess power for each RMST estimator as t^* varies for 10,000 simulations.

	Non-parametric estimator				Correctly specified parametric estimator				Misspecified parametric estimator			
β_3	Z	$\Delta(\hat{\beta}_3)$	s.e($\Delta(\hat{\beta}_3)$)	Power	Z	$\Delta(\hat{\beta}_3)$	s.e($\Delta(\hat{\beta}_3)$)	Power	Z	$\Delta(\hat{\beta}_3)$	s.e($\Delta(\hat{\beta}_3)$)	Power
**-2.00**	2.08	13.83	6.70	0.58	2.59	13.90	5.40	0.77	2.14	12.42	5.83	0.61
**-1.79**	2.17	14.47	6.73	0.60	2.66	14.49	5.49	0.78	2.25	13.21	5.89	0.64
**-1.58**	2.28	15.33	6.78	0.63	2.76	15.30	5.59	0.80	2.39	14.20	5.96	0.68
**-1.37**	2.36	15.97	6.82	0.67	2.82	16.03	5.71	0.82	2.52	15.12	6.02	0.73
**-1.16**	2.51	17.04	6.85	0.71	2.93	17.01	5.84	0.84	2.69	16.33	6.10	0.78
**-0.95**	2.64	18.04	6.89	0.75	3.04	18.05	5.98	0.85	2.86	17.54	6.17	0.82
**-0.74**	2.81	19.23	6.92	0.78	3.17	19.24	6.12	0.88	3.04	18.87	6.25	0.85
**-0.53**	2.96	20.36	6.96	0.82	3.29	20.42	6.25	0.90	3.22	20.22	6.31	0.88
**-0.32**	3.11	21.47	6.97	0.86	3.42	21.57	6.35	0.91	3.40	21.48	6.36	0.91
**-0.11**	3.25	22.46	6.98	0.88	3.54	22.51	6.42	0.93	3.53	22.47	6.40	0.93
**0.11**	3.37	23.24	6.98	0.90	3.65	23.32	6.44	0.94	3.66	23.3	6.42	0.94
**0.32**	3.47	23.87	6.96	0.91	3.76	23.90	6.40	0.95	3.74	23.85	6.42	0.95
**0.53**	3.52	24.13	6.94	0.92	3.86	24.13	6.30	0.96	3.79	24.06	6.41	0.95
**0.74**	3.48	23.9	6.93	0.92	3.90	23.80	6.15	0.96	3.75	23.75	6.38	0.95
**0.95**	3.39	23.27	6.93	0.91	3.92	23.13	5.94	0.97	3.67	23.12	6.35	0.93
**1.16**	3.25	22.29	6.93	0.89	3.91	22.16	5.69	0.97	3.57	22.37	6.31	0.92
**1.37**	3.07	21.20	6.96	0.86	3.91	21.07	5.40	0.97	3.47	21.56	6.27	0.90
**1.58**	2.86	19.87	7.01	0.80	3.89	19.82	5.11	0.97	3.35	20.70	6.23	0.87
**1.79**	2.65	18.58	7.07	0.74	3.84	18.46	4.81	0.97	3.24	19.88	6.19	0.84
**2.00**	2.43	17.25	7.13	0.68	3.83	17.25	4.51	0.97	3.13	19.04	6.15	0.82

Table S4: Mean simulated Z-value, treatment effect, and associated standard error to assess power for each RMST estimator as β_3 varies for 10,000 simulations.

t^*	Non-parametric estimator Z	$\Delta(\hat{t}^*)$	s.e$(\Delta(\hat{t}^*))$	TIE	Correctly specified parametric estimator Z	$\Delta(\hat{t}^*)$	s.e$(\Delta(\hat{t}^*))$	TIE	Misspecified parametric estimator Z	$\Delta(\hat{t}^*)$	s.e$(\Delta(\hat{t}^*))$	TIE
**1.00**	-0.01	0.00	0.01	0.000	0.00	0.00	0.00	0.023	0.00	0.00	0.00	0.026
**7.26**	0.01	0.00	0.22	0.025	-0.01	0.00	0.08	0.024	-0.01	0.00	0.07	0.026
**13.53**	0.00	0.00	0.56	0.027	0.00	0.00	0.24	0.026	0.00	0.00	0.22	0.026
**19.79**	0.00	0.00	0.95	0.031	0.01	0.00	0.49	0.027	0.00	0.00	0.45	0.029
**26.05**	0.00	0.00	1.40	0.027	0.00	0.00	0.80	0.028	0.01	0.01	0.75	0.029
**32.32**	0.02	0.03	1.87	0.028	0.00	0.00	1.16	0.024	0.00	0.00	1.11	0.024
**38.58**	0.00	0.00	2.36	0.030	0.00	0.01	1.56	0.029	0.01	0.01	1.50	0.032
**44.84**	0.00	0.00	2.87	0.028	0.00	0.00	1.99	0.030	0.00	0.01	1.95	0.035
**51.11**	0.00	0.01	3.37	0.028	0.00	0.00	2.44	0.029	0.00	0.00	2.42	0.031
**57.37**	0.00	0.00	3.90	0.028	-0.01	-0.02	2.92	0.029	-0.01	-0.03	2.92	0.030
**63.63**	0.01	0.04	4.41	0.032	0.01	0.03	3.41	0.029	0.01	0.03	3.44	0.031
**69.89**	0.00	-0.01	4.93	0.026	0.00	0.01	3.91	0.028	0.00	0.00	3.98	0.030
**76.16**	0.01	0.04	5.44	0.027	0.01	0.03	4.41	0.028	0.00	0.01	4.53	0.030
**82.42**	-0.01	-0.05	5.96	0.028	0.00	-0.02	4.92	0.029	-0.01	-0.04	5.09	0.029
**88.68**	0.00	-0.02	6.46	0.027	0.00	0.02	5.43	0.028	0.00	-0.01	5.66	0.030
**94.95**	0.01	0.07	6.96	0.028	0.01	0.06	5.94	0.029	0.01	0.06	6.23	0.033
**101.21**	0.00	0.01	7.45	0.029	0.01	0.04	6.44	0.029	0.00	0.02	6.79	0.032
**107.47**	0.00	0.01	7.93	0.029	0.00	0.00	6.94	0.031	0.00	-0.04	7.36	0.033
**113.74**	0.01	0.09	8.41	0.028	0.01	0.04	7.44	0.029	0.00	0.02	7.93	0.029

Table S5: Mean simulated Z-value, treatment effect, and associated standard error to assess Type I error (TIE) rate for each RMST estimator as t^* varies for 10,000 simulations.

β_3	Non-parametric estimator Z	$\Delta(\hat{\beta}_3)$	s.e($\Delta(\hat{\beta}_3)$)	TIE	Correctly specified parametric estimator Z	$\Delta(\hat{\beta}_3)$	s.e($\Delta(\hat{\beta}_3)$)	TIE	Misspecified parametric estimator Z	$\Delta(\hat{\beta}_3)$	s.e($\Delta(\hat{\beta}_3)$)	TIE
**-2.00**	0.00	-0.02	7.30	0.029	-0.01	-0.04	5.64	0.023	0.00	-0.02	6.38	0.031
**-1.79**	-0.01	-0.04	7.32	0.028	-0.01	-0.05	5.76	0.029	-0.01	-0.04	6.44	0.032
**-1.58**	-0.01	-0.05	7.34	0.027	0.00	0.00	5.90	0.028	0.00	-0.02	6.51	0.029
**-1.37**	0.01	0.10	7.35	0.029	0.01	0.09	6.06	0.030	0.02	0.13	6.58	0.033
**-1.16**	0.00	0.03	7.36	0.029	0.00	0.01	6.22	0.026	0.01	0.05	6.64	0.029
**-0.95**	0.02	0.11	7.35	0.027	0.01	0.07	6.38	0.030	0.01	0.07	6.70	0.032
**-0.74**	0.00	-0.03	7.32	0.029	0.00	0.02	6.52	0.030	0.01	0.04	6.73	0.036
**-0.53**	0.00	0.01	7.27	0.027	0.00	-0.01	6.63	0.029	0.00	-0.01	6.74	0.028
**-0.32**	-0.01	-0.04	7.18	0.028	-0.01	-0.04	6.69	0.028	-0.01	-0.04	6.72	0.030
**-0.11**	0.00	0.00	7.07	0.028	-0.01	-0.06	6.68	0.027	-0.01	-0.05	6.67	0.027
**0.11**	0.00	0.01	6.92	0.026	0.00	-0.01	6.60	0.026	0.00	0.00	6.58	0.026
**0.32**	0.00	0.00	6.77	0.029	0.00	-0.03	6.42	0.029	0.00	-0.02	6.46	0.029
**0.53**	0.01	0.09	6.61	0.027	0.01	0.07	6.17	0.028	0.01	0.08	6.33	0.030
**0.74**	-0.01	-0.03	6.49	0.026	-0.01	-0.03	5.86	0.026	0.00	-0.01	6.19	0.032
**0.95**	-0.01	-0.05	6.39	0.026	-0.01	-0.07	5.51	0.025	0.00	-0.01	6.04	0.035
**1.16**	0.01	0.07	6.36	0.027	0.02	0.09	5.15	0.025	0.01	0.08	5.91	0.042
**1.37**	0.01	0.08	6.36	0.027	0.00	0.01	4.82	0.026	0.02	0.10	5.79	0.050
**1.58**	0.01	0.04	6.41	0.028	-0.01	-0.06	4.52	0.025	0.00	0.01	5.68	0.060
**1.79**	0.00	-0.02	6.45	0.022	0.00	-0.02	4.26	0.024	0.00	-0.02	5.56	0.058
**2.00**	0.00	-0.02	6.51	0.025	0.00	0.01	4.05	0.025	0.00	0.01	5.47	0.069

Table S6: Mean simulated Z-value, treatment effect, and associated standard error to assess Type I error (TIE) rate for each RMST estimator as β_3 varies for 10,000 simulations.

6 Graphical Z-value breakdown

Relevant to the Results section of the main manuscript, below we present figures of the mean standard error, treatment effect, and Z-values used to assess power and type I error as t^* and β_3 varied.

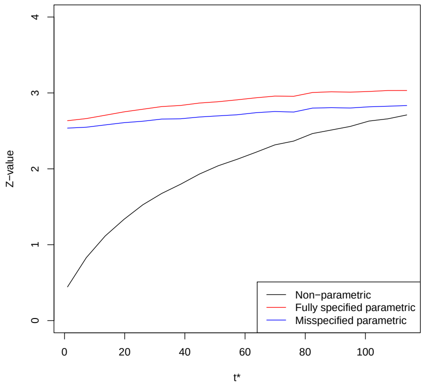

(a) Mean Z-value for varying t^*.

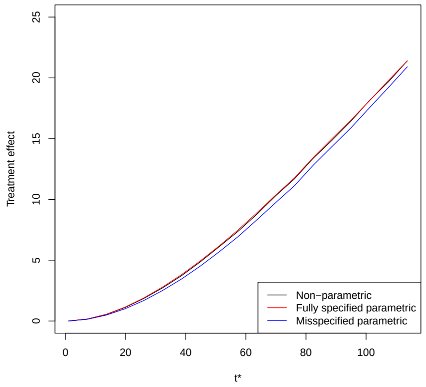

(b) Mean treatment effect for varying t^*.

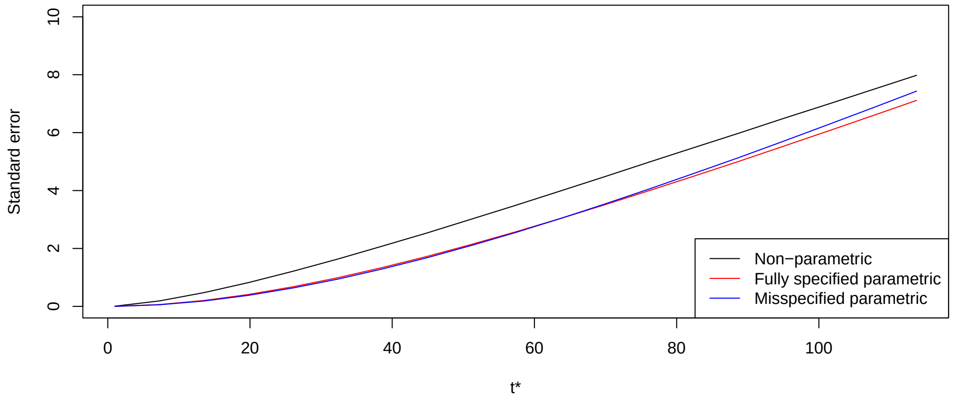

(c) Mean standard error for varying t^*.

Fig. S4: Mean simulated Z-value, treatment effect, and associated standard error to assess power for each RMST estimator as t varies for 10,000 simulations.

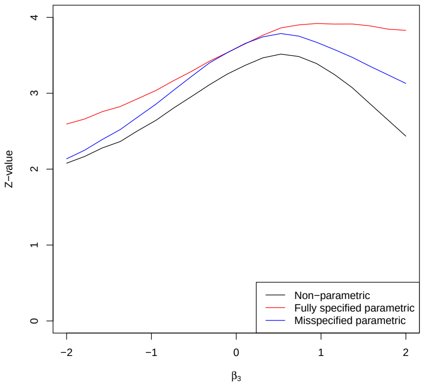

(a) Mean Z-value for varying β_3.

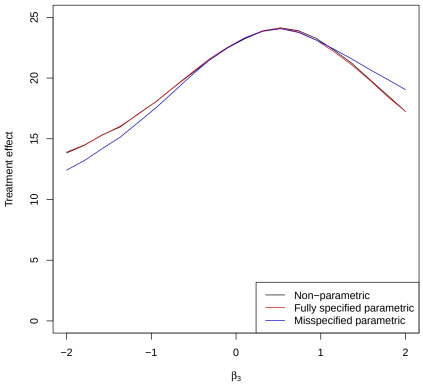

(b) Mean treatment effect for varying β_3.

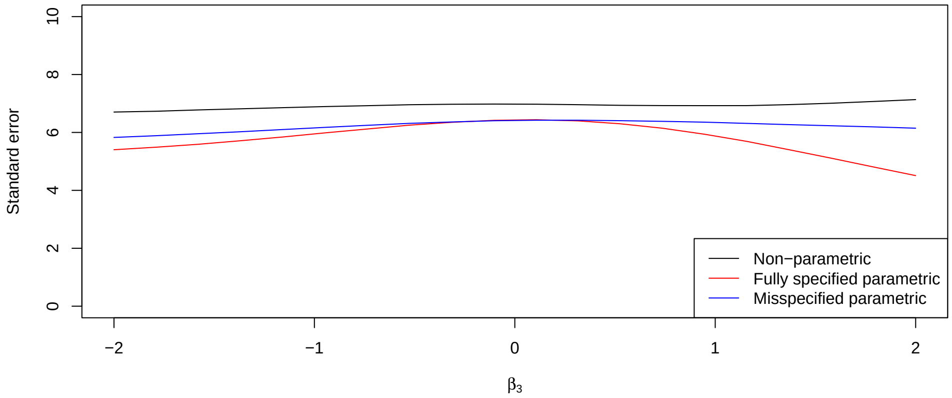

(c) Mean standard error for varying β_3.

Fig. S5: Mean simulated Z-value, treatment effect, and associated standard error to assess power for each RMST estimator as β_3 varies for 10,000 simulations.

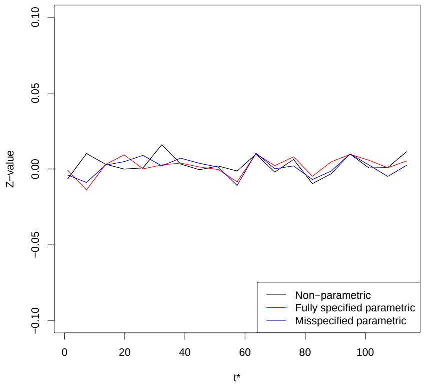

(a) Mean Z-value for varying t^*.

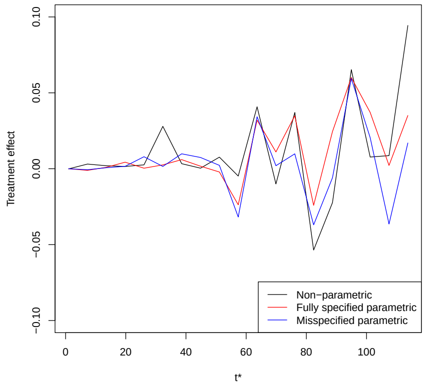

(b) Mean treatment effect for varying t^*.

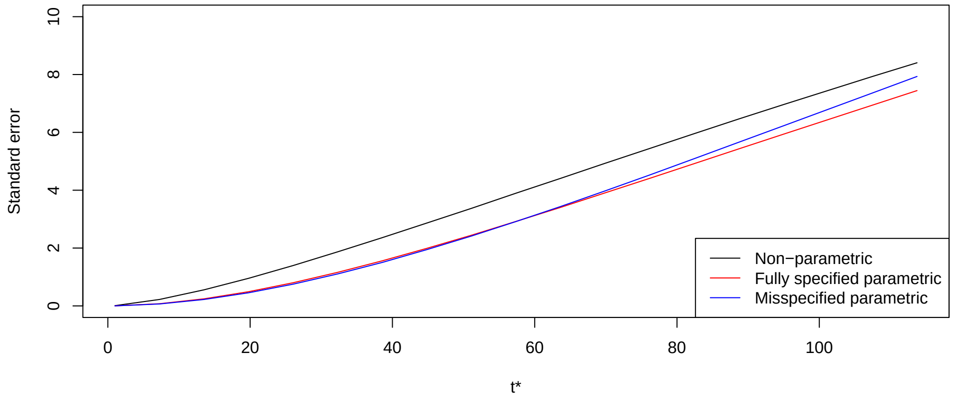

(c) Mean standard error for varying t^*.

Fig. S6: Mean simulated Z-value, treatment effect, and associated standard error to assess type I error for each RMST estimator as t varies for 10,000 simulations.

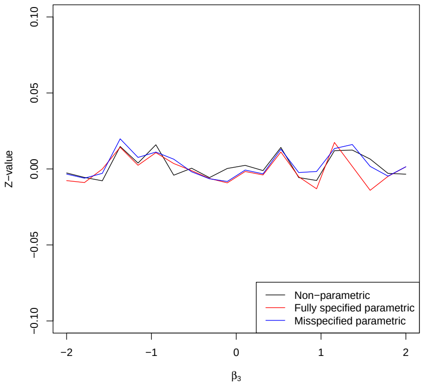

(a) Mean Z-value for varying β_3.

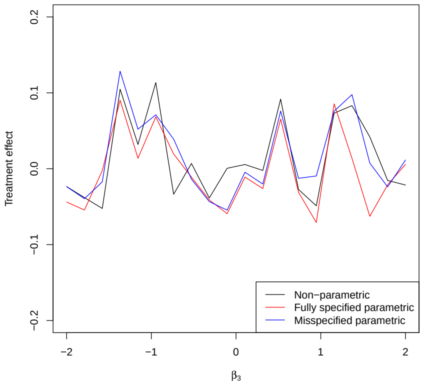

(b) Mean treatment effect for varying β_3.

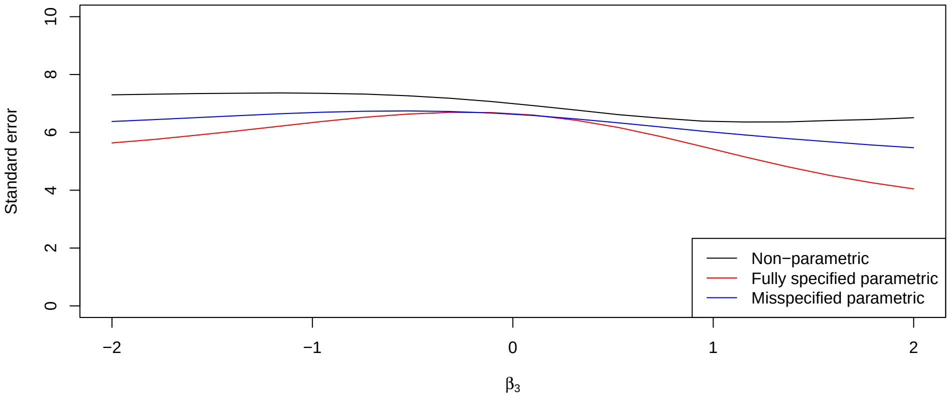

(c) Mean standard error for varying β_3.

Fig. S7: Mean simulated Z-value, treatment effect, and associated standard error to assess type I error for each RMST estimator as β_3 varies for 10,000 simulations.

7 Crossing survival curves in the alternative direction

We investigate the effects of varying t^* when RMST is used to analyse a trial with crossing survival curves. Initially the control is considered more effective than the experimental treatment but then becomes less effective at a knot point and the survival curves cross. The curves cross in the opposite direction than in the main text and the results are obtained using the same simulation results but by permuting the treatment labels. Figure S8 shows an example Kaplan-Meier plot such as this trial.

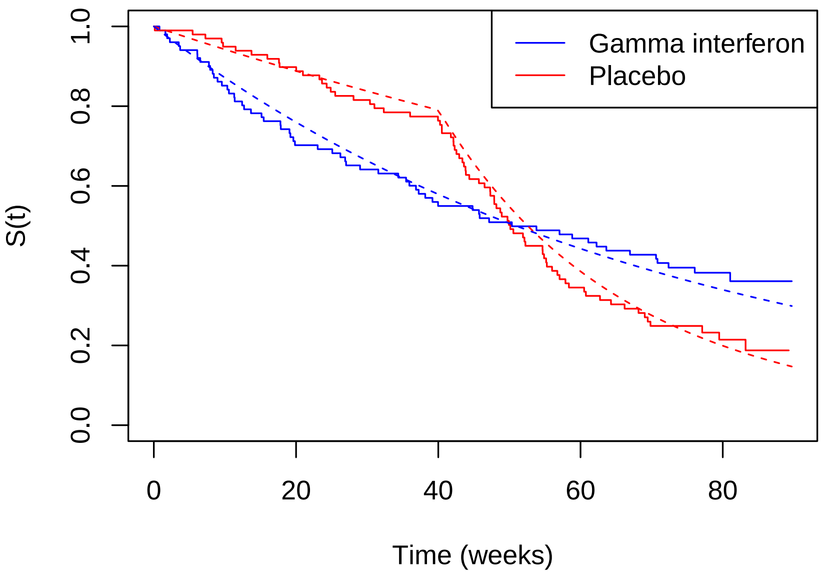

Fig. S8: A Kaplan-Meier plot showing the survival of 200 patients receiving either Gamma interferon or control treatment for survival distribution with crossing survival curves. Dashed lines show the parametric value of the survival function.

Figure S9 shows the results of this simulation study. We see that the type I error rates are unaffected but the power is highly susceptible to changes in t^*. As t^* increases beyond the crossing point at approximately 50 weeks, the power of the trial increases

as the experimental treatment becomes more effective. In general, power is low at approximately 10% for t^* at 120 weeks because the treatment is initially not working as well as placebo. Under this model, the misspecified parametric endpoint appears more powerful than the correctly specified parametric endpoint. The misspecified knot point $\tilde{t}_1 = 50$ is later than the true knot point $t_1 = 40$. This means that the survival probabilities are underestimated on the placebo arm and the difference between treatment is smaller.

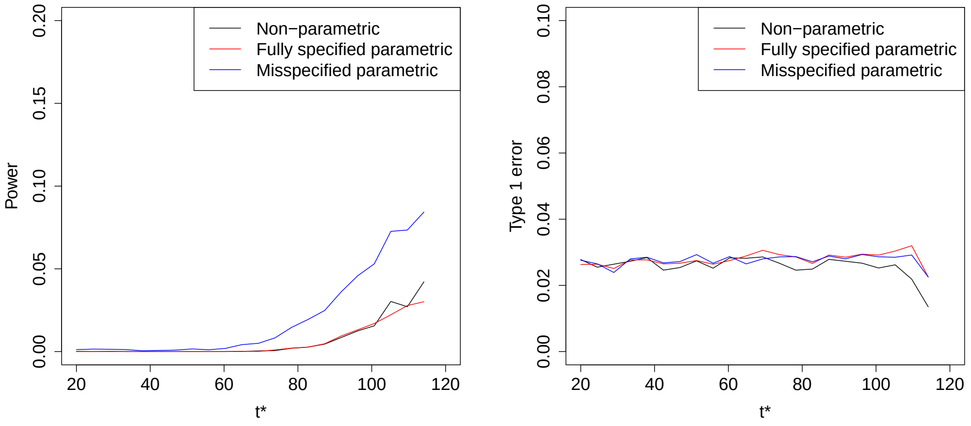

Fig. S9: Simulation study results showing type I error rates and power when data is fit to a Cox proportional hazards model with crossing survival curves. Varying t^* with true fixed knot-point $t_1 = 40$ and misspecified knot-point $\tilde{t}_1 = 50$.
